# TLR Stimulation of Bone Marrow Lymphoid Precursors from Childhood Acute Leukemia Modifies Their Differentiation Potentials

**DOI:** 10.1155/2013/846724

**Published:** 2013-09-10

**Authors:** Elisa Dorantes-Acosta, Eduardo Vadillo, Adriana Contreras-Quiroz, Juan Carlos Balandrán, Lourdes Arriaga-Pizano, Jessica Purizaca, Sara Huerta-Yepez, Elva Jiménez, Wendy Aguilera, Aurora Medina-Sanson, Héctor Mayani, Rosana Pelayo

**Affiliations:** ^1^Oncology Research Unit, Oncology Hospital, Mexican Institute for Social Security, Avenue Cuauhtemoc 330, Colonia Doctores, 06720 Mexico City, Mexico; ^2^Federico Gómez Children's Hospital, 06720 Mexico City, Mexico; ^3^Medical Sciences Program, National Autonomous University of Mexico, 04510 Mexico City, Mexico; ^4^National School of Biological Sciences, National Polytechnic Institute, 11340 Mexico City, Mexico; ^5^Molecular Biomedicine Program, CINVESTAV, 07360 Mexico City, Mexico; ^6^Immunochemistry Research Unit, Medical Specialties Hospital, Mexican Institute for Social Security, 06720 Mexico City, Mexico; ^7^Moctezuma Children's Hospital, 15340 Mexico City, Mexico; ^8^General Hospital, La Raza Medical Center, Mexican Institute for Social Security, 02990 Mexico City, Mexico

## Abstract

Acute leukemias are the most frequent childhood malignancies worldwide and remain a leading cause of morbidity and mortality of relapsed patients. While remarkable progress has been made in characterizing genetic aberrations that may control these hematological disorders, it has also become clear that abnormalities in the bone marrow microenvironment might hit precursor cells and contribute to disease. However, responses of leukemic precursor cells to inflammatory conditions or microbial components upon infection are yet unexplored. Our previous work and increasing evidence indicate that Toll-like receptors (TLRs) in the earliest stages of lymphoid development in mice and humans provide an important mechanism for producing cells of the innate immune system. Using highly controlled co-culture systems, we now show that lymphoid precursors from leukemic bone marrow express TLRs and respond to their ligation by changing cell differentiation patterns. While no apparent contribution of TLR signals to tumor progression was recorded for any of the investigated diseases, the replenishment of innate cells was consistently promoted upon *in vitro* TLR exposure, suggesting that early recognition of pathogen-associated molecules might be implicated in the regulation of hematopoietic cell fate decisions in childhood acute leukemia.

## 1. Introduction

Acute leukemias (ALs) are characterized by the uncontrolled production of hematopoietic precursor cells of the lymphoid or myeloid series within the bone marrow (BM) and at present, stand as the most common cause of childhood cancer worldwide [1]. Of the two types of AL, acute lymphoblastic leukemia (ALL) shows the highest frequency, accounting for 85% of the cases, while acute myeloid leukemia (AML) constitutes 15% of them [2]. Nearly 85% of ALL cases have a precursor B-cell immunophenotype and approximately 15% show a T-cell phenotype. Congenital leukemia and mixed-lineage leukemia are rare disease entities that are associated with a poor prognosis and must be distinguished from typical ALL or AML. Congenital leukemia represents only 3% of AL, whereas mixed-lineage leukemias, possessing characteristics of both lymphoid and myeloid precursor cells, are 2% of AL in children [3]. 

Although important breakthroughs in the investigation of genetic pathogenesis of acute leukemias and new treatment strategies have been recorded in the last few years [4, 5], our understanding of the mechanisms that damage the early programs of hematopoietic development remains poor [6]. Even when cancer stem cells (CSCs) in myeloid leukemia have been strictly depicted as the cells responsible for tumor maintenance, the cellular origin of ALL is still a fundamental matter in question [7, 8]. Moreover, the complexity of leukemogenesis increases when considering the influence of the BM microenvironment in the very early fate decisions of the hematopoietic development [9].

According to the classical model of hierarchical hematopoiesis, blood cells arise from a unique cell population of hematopoietic stem cells (HSCs) that reside in specialized niches within the BM and progress through critical stages of differentiation to multipotent early progenitors. The culminating production of myeloid and lymphoid cells involves a stepwise maturation of lineage-committed precursor cells and the concerted action of transcription factors, cell to cell interactions, growth factors, and cytokines that regulate the expression or silencing of differentiation genes [10, 11]. Interestingly, recent reports indicate that seminal cells in BM are able to recognize microbial/viral components, and the effect of this stimulation is the redirection of differentiation fates during formation of hematopoietic cells [12–17], suggesting that pattern-recognition receptors are involved in the most primitive stages of hematopoiesis and contribute to emergent cell replenishment. 

The functional expression of Toll-like receptors (TLRs) in the hematopoietic stem and progenitor cells was first reported by Nagai and colleagues [18], and their striking response to TLR ligation by proliferation and differentiation toward the myeloid lineage bypassing exogenous growth factors entailed TLRs to hematopoiesis and also revolutionized our understanding of the mechanisms governing infection responses [11]. We then discovered that lymphoid progenitors in Herpes-infected mice become polarized to a different fate in a TLR9-dependent fashion [19, 20]. The described phenomena in mice can be extrapolated to humans and seminal cells which posses unique TLR expression patterns that make them vulnerable to extrinsic or endogenous TLR signals, responding with immediate innate immune cell production [11]. Of note, early multilymphoid progenitors from adult BM react to TLR ligation by upregulating expression of cytokine receptors and accelerating the production of functional natural killer (NK) cells (Vadillo et al., submitted). 

In hematological abnormalities such as acute leukemias, increasing evidence suggests the prevalence of inflammatory environments, along with recurrent infections and chemotherapy-associated damage, strengthening the possibility that TLR-expressing primitive cells represent the beginning of instability of the lineage [21, 22]. Whether these signals contribute to disease or promote lineage conversion in relapsed leukemia are currently topical questions.

We report here that lymphoid precursor cells from different subtypes of acute leukemia, including acute lymphoblastic leukemia, congenital leukemia, mixed-lineage leukemia, and acute myeloid leukemia express distinct and heterogeneous profiles of TLR transcripts and respond to stimulation by TLR agonists, thereby redirecting their differentiation potentials and increasing the production of myeloid and NK cells. Thus, the competence of lymphoid progenitors to boost the generation of emerging innate cells during infection in the setting of acute leukemia is apparently unimpaired, and no obvious input to tumor progression was documented in our controlled culture systems. TLR ligation on lymphoid progenitor cells may provide a secondary strategy to improve cancer surveillance in childhood leukemia. 

## 2. Materials and Methods

### 2.1. Collection of Hematopoietic Cells

 Bone marrow (BM) specimens, collected according to international and institutional guidelines, were obtained through BM aspiration from 16 pediatric patients diagnosed with acute leukemia (AL), before any treatment. Among them, 10 patients fulfilled the standard diagnostic criteria for B-cell precursor acute lymphoblastic leukemia (B-ALL), 1 for congenital leukemia (C-ALL), 2 for T-cell acute lymphoblastic leukemia (T-ALL), 2 for mixed acute leukemia (M-ALL), and 1 for acute myeloid leukemia (AML) [1]. As a control, one bone marrow sample was also obtained from a healthy child undergoing minor orthopedic surgery. All procedures were approved by the Ethics, Research, and Biosafety Committee of the Federico Gómez Children's Hospital (Registry HIM/2009/033) and by the National Committee of Scientific Research at the Mexican Institute for Social Security (Registry R-2010-785-012) in Mexico City. Samples were collected after informed consent from the parents. The general characteristics of the included patients are summarized in Table 1. The median blast infiltration of patients' BM at the time of diagnosis was 89%.

### 2.2. Isolation of Precursor Cells

Mononuclear cells (MNCs) from AL patients were prepared by Ficoll-Paque Plus (GE Healthcare Bioscience) gradient separation. CD34^+^ cells containing hematopoietic stem and progenitor cells were enriched from MNCs using the Human CD34 Progenitor Cell Isolation Kit (Miltenyi Biotec) according to the manufacturer's instructions. When the CD34^+^ cell numbers were enough for the conduction of most experiments plus an additional staining, the purity was confirmed by flow cytometry. The mean (average) value of the obtained CD34^+^ cell numbers was 26 × 10^6^ ± 41.70 × 10^6^, while the same value for calculated cell frequencies was 18.5 × 10^6^ ± 22.2 × 10^6^.

### 2.3. Reverse-Transcription Polymerase Chain Reaction (RT-PCR) Analysis of Gene Expression

To determine the expression of TLR1–10 transcripts in BM CD34^+^ cells from leukemic patients, mRNAs were isolated with Trizol (Invitrogen) and cDNAs were prepared using Moloney murine leukemia virus reverse transcriptase (Invitrogen). The PCR was performed using ampli-Taq DNA polymerase with a step program of denaturation (94°C) for 30 seconds, annealing (60°C) for 30 seconds and elongation (72°C) for 1 minute. PCR products were separated in a 1% (w/v) agarose gel, and visualized by staining with ethidium bromide. A collection of TLR1–10 primers was provided by InvivoGen, and 18 s rRNA subunit was used as housekeeping gene (Invitrogen). For investigation of the lineage transcription factor (TF) expression after 4-week lymphoid cultures, primers for PAX-5 (B-cell related TF), EBP*α* (myeloid-related TF), and Id2 (NK cell-related TF) transcripts were synthesized by Sigma. 

### 2.4. TLR Stimulation

Primitive CD34^+^ precursor cells from AL patients were cultured for 48 hours in *α*-MEM serum-free culture medium with ligands agonists for TLR1/2 (Pam3CSK4), TLR3 (PolyI:C), TLR4 (LPS), TLR5 (Flagellin), TLR2/6 (MALP2), TLR8 (PolyU), and TLR9 (a mix of CpG-containing oligonucleotides, CpG-ODN, A, and B) (InvivoGen). Cells were further cocultured with MS5 stromal cells for 30 days. 

### 2.5. Cytokine Detection

Supernatants were collected after TLR4, TLR5, TLR8, and TLR9 stimulation's with their agonists LPS, flagellin, PolyU, and CpG, respectively. Supernatants were stored at −80°C until analysis. The cytokine, chemokine, and growth factor content in supernatants was measured by Multiplex Milliplex Map Immunoassay (Merck Millipore), following the manufacturer's recommended protocols. The assay included EGF, G-CSF, GM-CSF, IFN*α*, IFN*γ*, IL-10, IL-12 p70, IL-17, IL-1*β*, IL-2, IL-4, IL-6, IL-8, MCP-1, RANTES, and VEGF.

### 2.6. Stromal Cell Cocultures

Upon exposure of BM precursors to TLR agonists, cells were transferred to an MS-5 stromal cell monolayer and cocultured with them for 30 days in lymphoid conditions, according to a modified previous report [23]. The *α*-modified essential medium (*α*-MEM) was supplemented with 10% fetal bovine serum, 1 ng/mL Flt3-L (FL), 2 ng/mL SCF, 5 ng/mL IL-7, and 10 ng/mL IL-15 (Preprotech) and contained 100 U/mL penicillin & 100 mg/mL streptomycin. Coculture systems were incubated at 37°C in a humidified atmosphere of 5% CO_2_. This controlled system promotes the efficient differentiation of hematopoietic stem, progenitor, and precursor cells towards B cells, NK cells, and lymphoid-related dendritic cells. Unstimulated mock cells were cultured in the same experiment for comparison.

### 2.7. Immunophenotyping by Flow Cytometry

Phenotyping of hematopoietic cells from lymphoid cocultures was performed by four-color and five-color flow cytometry on a FACSCalibur flow cytometer (BD Biosciences) and on a CyAn flow cytometer (Beckman Coulter), respectively. Cells were enumerated after culture to calculate cell frequencies and yields per input progenitor prior to staining with directly conjugated fluorescent antibodies (Invitrogen and BD Pharmingen). Precursor cells were identified as CD34^+^ CD19^−^ CD14^−^, whereas newly produced B cells were CD34^+/−^ CD19^+^ CD14^−^, and myeloid-related cells were CD34^−^ CD19^−^ CD14^+^. Cells expressing CD56 and CD11c were considered mature NK cells, while CD56^+^CD11c^−^ cells were classified as differentiating NK cells. Analysis of flow cytometry data was performed using the FlowJo 7.6.1 software, and final yield per input values were calculated on the basis of specific lineage cell frequencies within each condition. 

### 2.8. Cytotoxicity Assay

 NK cell cytolytic activity was performed using a modification of the Guava EasyCyte CellToxicity Assay (Guava 4500-0230). Briefly, K562 cell line (ATCC) was kept in log phase growth in RPMI 1640 supplemented with 10% of fetal calf serum (FCS). K562 cells were stained with 5 *μ*M CFSE. *In vitro* differentiated NK cells from ALL progenitor cells were enumerated by flow cytometry and mixed with CFSE labeled K562 cells using 10 : 1 effector to target ratio and incubated for 4 hours at 37°C. IL-2 (40 ng/mL) (Preprotech) was added to induce NK cell activation. After incubation, cells were washed and 7-AAD (BD Pharmigen) incorporated. Flow cytometry (Cyan Beckman Coulter) was conducted to determine functionality.

### 2.9. Statistics

The Prism software, version 5.01 (GraphPad) was used for statistical analysis. Comparisons between groups were performed with the unpaired *t* test. *P* values were 2-tailed and considered significant if they are less than 0.05.

## 3. Results and Discussion

### 3.1. Hematopoietic Precursors from Acute Leukemia Variably Express Functional TLRs and Respond to Its Ligation with Cytokine Production

Pathogen recognition by cells of the immune system is carried out by a growing list of pattern-recognition receptors, including Toll-like receptors, which start innate immune responses by inducing the production of proinflammatory cytokines and the expression of co-stimulatory molecules [24–27]. The recent discovery of regulation of the hematopoietic developmental pathways by TLRs suggests that they are involved in the early cell fate decisions and contribute to the emergent replenishment of innate immune cells during infections [18–20, 28–30]. Our work now provides relevant information about cell responses to environmental cues such as pathogen components in the setting of hematological malignancies. We show that B-cell differentiation potential of lymphoid precursors from most AL subtypes is stable upon TLR stimulation. In contrast, these primitive cells efficiently increase the production of myeloid and NK innate cells as a result of TLR signaling. 

**Figure 1:**
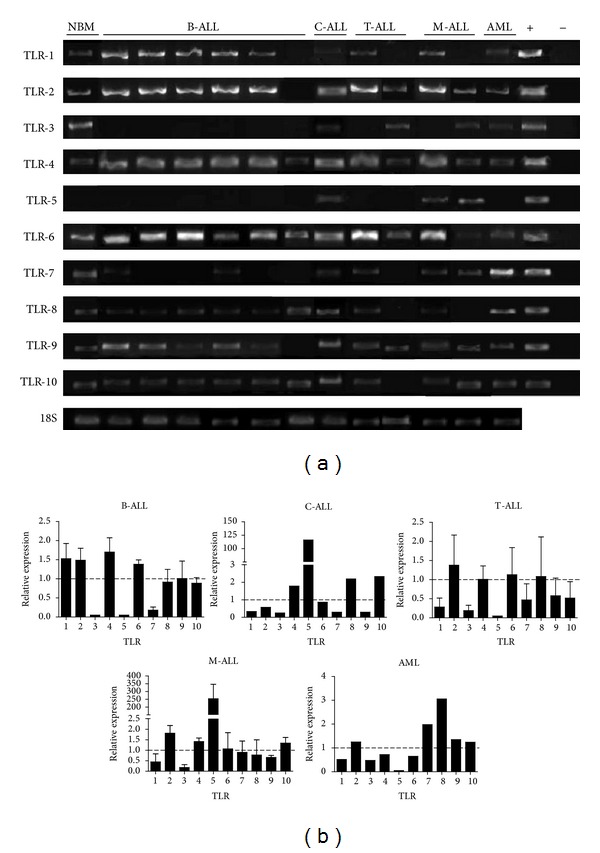
Bone marrow-derived precursor cells from childhood acute leukemia express Toll-like receptors (TLRs). Bone marrow CD34^+^ cells were purified by magnetic separation and analyzed for TLRs transcription by RT-PCR (a). Positive controls resulted from TLR-expressing plasmids, while the negative control was DEPC H_2_O. Transcript expression levels in various acute leukemia subtypes relative to their normal counterpart were tabulated after density analyses (b). 18 s rRNA subunit was used as housekeeping gene. NBM: normal bone marrow; B-ALL: B-cell precursor acute lymphoblastic leukemia; C-ALL: congenital acute lymphoblastic leukemia; T-ALL: T-cell acute lymphoblastic leukemia; M-ALL: mixed acute lymphoblastic leukemia; AML: acute myeloid leukemia.

 To determine the expression of TLR transcripts, we performed reverse-transcriptase PCR (RT-PCR) analysis on BM CD34^+^ cells from 5 subtypes of acute leukemia: B-cell precursor acute lymphoblastic leukemia (B-ALL), congenital leukemia (C-ALL), T-cell acute lymphoblastic leukemia (T-ALL), mixed acute leukemia (M-ALL), and acute myeloid leukemia (AML) (Figure 1). While TLR transcripts were variably expressed by pediatric leukemia precursors, cells from all sources showed detectable levels. We and other groups have previously reported certain qualitative discrepancies in TLR expression within BM compartment, depending on the TLR in question, suggesting substantial heterogeneity based on the source of sample (Vadillo et al., submitted), and the category of early hematopoietic progenitors within it [13, 14, 31]. Moreover, patterns of TLR transcript expression may vary between differentiating progenitors with their effective display occurring while differentiation progresses [20, 32]. Our current results suggest that leukemia subtypes also possess different patterns of TLR transcript expression (Figure 1(a)). Strikingly, TLR3 transcripts could not be detected in B-ALL, and its expression is extremely low in any of the other investigated diseases (Figure 1(b)). It is of importance to note that TLR5 expression was highly expressed by the poor prognosis congenital ALL and mixed leukemia. TLR2, 4, 7, and 9 showed distinct patters of TLR transcript expression and may differ from their normal counterparts. 

Although a possible role of TLR patterns in the pathogenesis of these diseases is still unclear, the observed variations could be explained by predominance of a conspicuous type of progenitor within each sample, enabling the possibility of having variable skills to fight infectious and neoplastic diseases.

**Figure 2:**
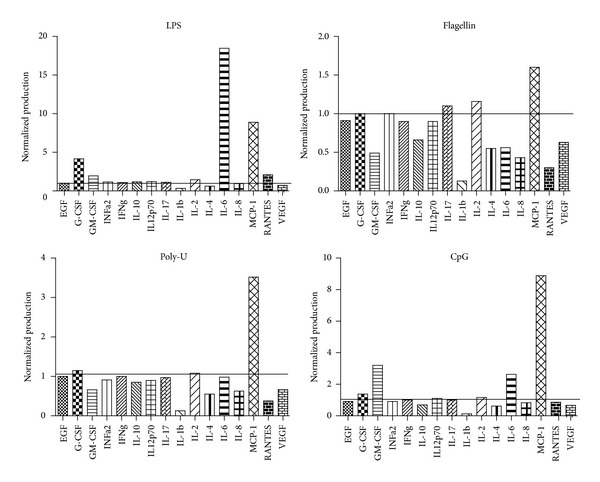
TLR agonists promote the production of hematopoietic factors by precursor cells from B-cell acute lymphoblastic leukemia (ALL). ALL BM CD34^+^ cells were stimulated with TLR agonists for 48 h. LPS was used as a ligand for TLR4, while flagellin was the ligand for TLR5, PolyU for TLR7/8, and CpG for TLR9. Supernatants were further collected upon 48 h stimulation and assayed for 16 cytokines, chemokines, and growth factors. Normalized production relative to mock conditions was tabulated.

Production of proinflammatory cytokines and growth factors, including TNF*α*, IL-12, IL-3, IL-6, IL-8, GM-CSF, and MCP-1, as a result of TLR signaling in normal primitive hematopoietic cells has been documented [11, 31, 33, 34]. As part of the hematopoietic microenvironment, these soluble factors contribute to lineage cell fate decisions under specific conditions. Therefore, cytokines and growth factors were quantified in supernatants derived from stimulated or not CD34^+^ cells from acute lymphoblastic leukemia. G-CSF, IL-6, GM-CSF, and MCP-1 were probed to increase upon TLR stimulation (Figure 2), confirming that, as in normal hematopoietic progenitors, leukemic precursor cells are supplied with functional TLRs and respond to TLR agonists by producing cytokines. It is worth mentioning that MCP-1 was highly overproduced under the four investigated conditions. It is widely known that MCP-1 is a chemokine with suppressive effects on normal hematopoiesis, which attracts monocytes to leukemic cells without the promotion of positive effects on monocyte cytotoxicity. Its upregulation as a consequence of TLR ligation could possibly promote survival, proliferation, and adhesiveness of leukemia cells and may represent a possible explanation of microenvironmental imbalances in hematological malignancies [35]. Furthermore, during chemotherapy, MCP-1 is upregulated in cerebrospinal fluid, suggesting that cell destruction and DAMPs release may activate the TLR program, resulting in critical MCP-1 release [36]. 

### 3.2. TLR Agonists Alter the AL Precursor Differentiation Potentials; Promoting Myeloid and NK Cell Development

**Figure 3:**
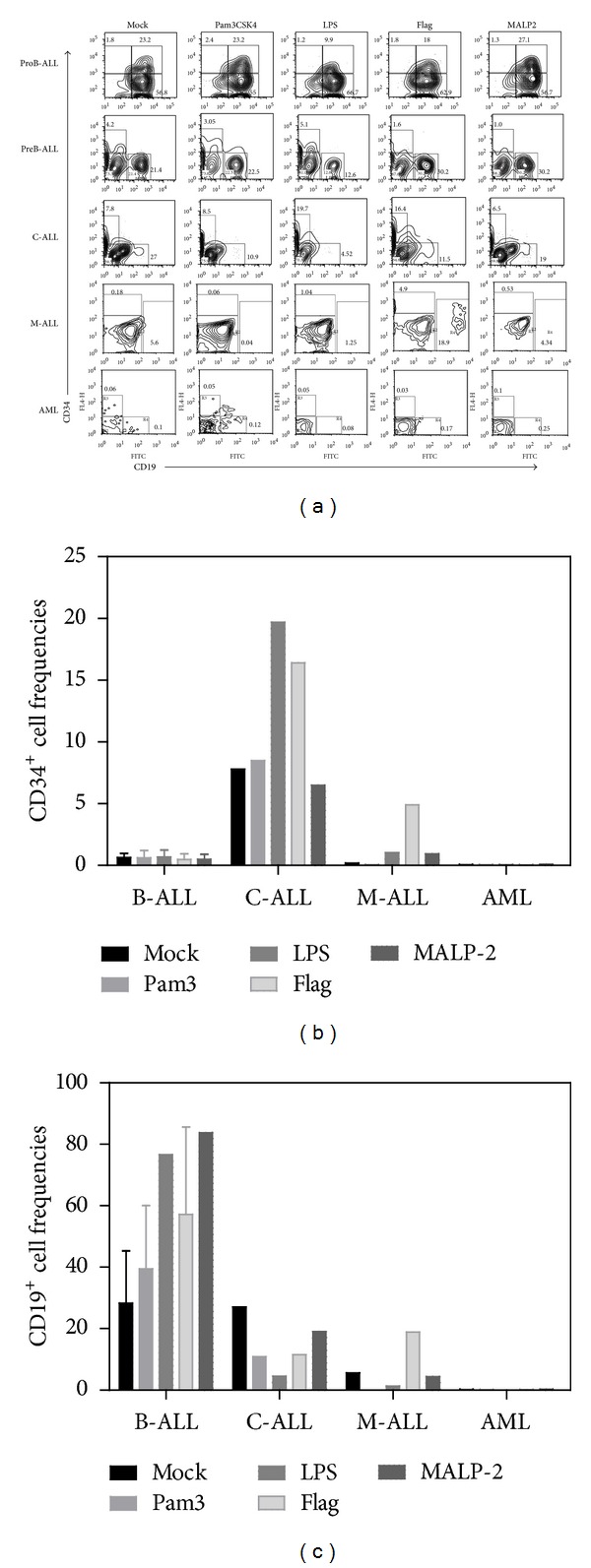
B-cell differentiation in acute leukemia is not crucially altered by membrane TLR ligation. BM CD34^+^ cells were purified from ProB-ALL, PreB-ALL, C-ALL, M-ALL, and AML and stimulated with Pam3CSK4 (TLR2 ligand), LPS (TLR4 ligand), Flagellin (TLR5 ligand), and MALP2 (TLR2/6 ligand) agonists for 48 h, followed by a 30 day-stromal cell coculture. Newly produced CD34^+^ and CD19^+^ cell fractions were further identified and enumerated by multiparametric flow cytometry (a). Cell frequencies within the culture for both CD34^+^ cells (b) and CD19^+^ B-lymphoid cells (c) are shown. ProB-ALL: B-cell precursor ALL with prevalence of CD34^+^CD10^+^CD19^+^ cells; PreB-ALL: B-cell precursor ALL with prevalence of CD3^−^CD10^+/−^CD19^+^ cells; C-ALL: congenital ALL; M-ALL: mixed ALL; AML: acute myeloid leukemia.

**Figure 4:**
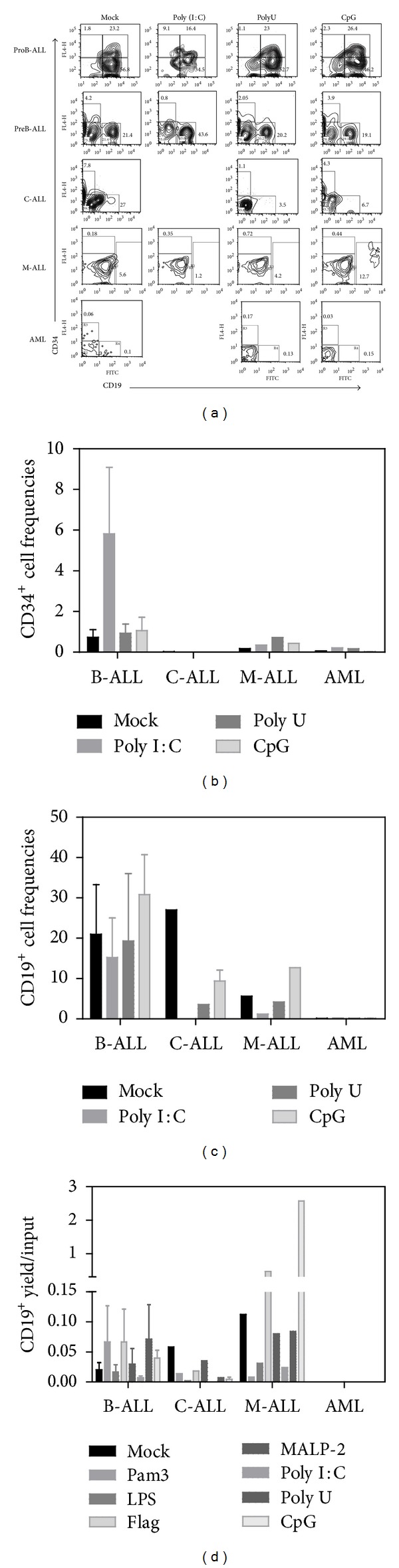
TLR9 contributes to *in vitro* production of B cells from mixed acute leukemia, but not from acute lymphoblastic leukemia-derived precursor cells. BM CD34^+^ cells were purified from ProB-ALL, PreB-ALL, C-ALL, M-ALL, and AML and stimulated with Poly(I:C) (TLR3 ligand), PolyU (TLR8 ligand), and CpG-ODN (TLR9 ligand) agonists for 48 h, followed by a 30 day-stromal cell coculture. Newly produced CD34^+^ and CD19^+^ cell fractions were further identified and enumerated by multiparametric flow cytometry (a). The indicated gates were used to discriminate cell populations. Cell frequencies within the culture for both CD34^+^cells (b) and CD19^+^ B-lymphoid cells (c) are shown. Total numbers of CD19^+^ recovered cells from each treatment condition were calculated and expressed as yields per input progenitor (d). ProB-ALL: B-cell precursor ALL with prevalence of CD34^+^CD10^+^CD19^+^ cells; PreB-ALL: B-cell precursor ALL with prevalence of CD3^−^CD10^+/−^CD19^+^ cells; C-ALL: congenital ALL; M-ALL: mixed ALL; AML: acute myeloid leukemia.

 To determine whether TLR signaling could rapidly influence hematopoietic progenitor cell fate decisions in leukemia, CD34^+^ BM fractions were highly purified and exposed to TLR agonist ligands for 48 hours. Further long-term cultures revealed that CD34^+^ cell production and/or maintenance was highly promoted in C-ALL when ligands for most membrane-associated TLRs were used (Figures 3(a) and 3(b)), suggesting that in the setting of some diseases such as C-ALL, TLRs may contribute to self-renewal of primitive cells. The same was true when B-ALL was exposed to the intracellular Poly(I:C) TLR ligand (Figures 4(a) and 4(b)). In contrast, B-cell production remained stable upon TLR treatment when compared to mock controls from most studied malignancies (Figures 3 and 4). Mixed acute leukemia primitive cells showed an interesting tendency to produce more B cells than the unstimulated counterparts after both flagellin and CpG treatment (Figures 3(a), 3(c), 4(a), and 4(d)). Flagellin has been recently shown to be radio-protective and to reduce graft versus host disease after transplantation through the promotion of functions in T-regulatory cells [37]. In spite of discrete changes in B cell frequencies within the stimulated cultures, none of the tested hematological diseases showed signs of tumor maintenance/progression in the yield per input values. 

**Figure 5:**
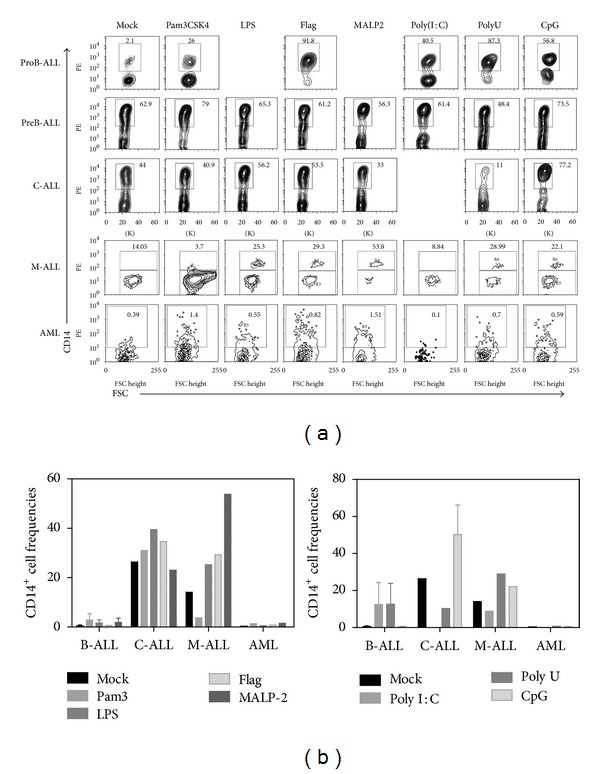
Development of CD14^+^ cells from lymphoid precursors is promoted by TLR ligation in acute lymphoblastic leukemias. BM CD34^+^ cells were purified from all investigated acute leukemia subtypes and stimulated with Pam3CSK4 (TLR2 ligand), LPS (TLR4 ligand), Flagellin (TLR5 ligand), MALP2 (TLR2/6 ligand), Poly(I:C) (TLR3 ligand), PolyU (TLR8 ligand), and CpG-ODN (TLR9 ligand) agonists for 48 h, followed by a 30 day-stromal cell coculture. Newly produced CD14^+^ cells were further identified and enumerated by multiparametric flow cytometry (a). The indicated gates were used to determine cell frequencies within the culture (b). Under these culture conditions, acute myeloid leukemia showed poor differentiation potentials and no apparent influence by TLR exposure. ProB-ALL: B-cell precursor ALL with prevalence of CD34^+^CD10^+^CD19^+^ cells; PreB-ALL: B-cell precursor ALL with prevalence of CD3^−^CD10^+/−^CD19^+^ cells; C-ALL: congenital ALL; M-ALL: mixed ALL; AML: acute myeloid leukemia.

In mice, it has been described that TLR2 and TLR4 ligations induce myeloid differentiation, a phenomenon that can also be achieved in humans when hematopoietic progenitors are stimulated with Pam3CSK4 and TLR7/8 ligands [18, 31]. In concordance, at the end of culture, the lymphoid precursors from all lymphoid acute leukemias changed the differentiation profile and produced higher frequencies of CD14-myeloid related cells (Figure 5). As expected, our controlled culture conditions do not promote regular myelopoiesis, and cells from AML BM were not robust in differentiation potential under the effect of these lymphoid environments. Myeloid cultures must be conducted to further evaluate their sensitivity to pathogen components. 

**Figure 6:**
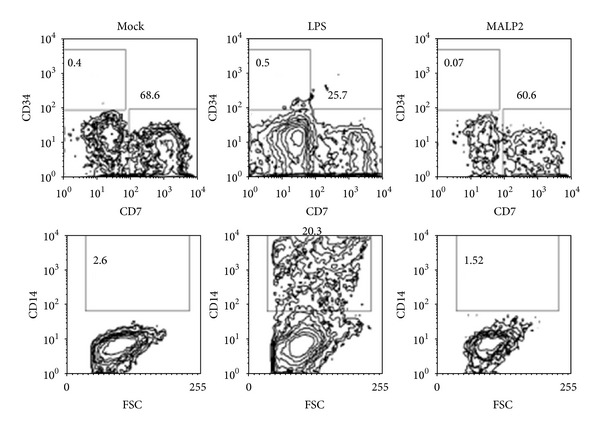
Rapid formation of CD14^+^ myeloid cells from T-ALL progenitors upon responsiveness to TLR4 signals. Long-term cultures (30 d) were conducted to assess CD14^+^ cell formation. While maintenance of CD7^+^ cells was unaltered, CD34^−^CD7^−^CD14^+^ tended to increase with the LPS treatment (middle panel).

The most powerful effect of LPS was recorded on the behavior of T-ALL precursors under lymphoid cultures, where CD14^+^ cells rapidly emerged (Figure 6).

**Figure 7:**
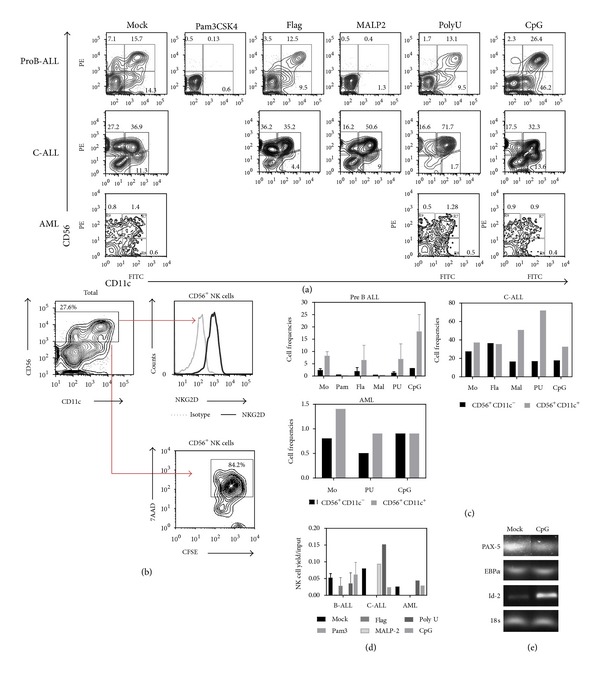
Intracellular TLRs lead to production of mature NK cell in both lymphoid and myeloid acute leukemia. ProB-ALL, C-ALL and AML CD34^+^ cells were purified and stimulated with Pam3CSK4 (TLR2 ligand), Flagellin (TLR5 ligand), MALP2 (TLR2/6 ligand), PolyU (TLR8 ligand) and CpG-ODN (TLR9 ligand) agonists for 48 h. After 30 day-culture, newly produced CD56^+^CD11c^−^ and CD56^+^CD11c^+^ developing and mature NK cells, respectively, were identified by multiparametric flow cytometry (a). CFSE^+^ K562 cells were used as target of cultured NK cells from Mock-ALL progenitors. Cytotoxicity was evaluated by 7AAD incorporation (b), and the expression of the activating receptor NKG2D analyzed by flow cytometry (b). The indicated quadrants in (a) were used to determine cell frequencies for each leukemia subtype (c). Total numbers of mature CD56^+^ recovered cells from the various treatment conditions were calculated and expressed as yields per input progenitor (d). The expression of PAX-5, EBP*α* and Id-2 transcription factors was analyzed by RT-PCR upon 48 h-CpG stimulation (e). ProB-ALL: B-cell precursor ALL with prevalence of CD34^+^CD10^+^CD19^+^ cells; PreB-ALL: B-cell precursor ALL with prevalence of CD3^−^CD10^+/−^CD19^+^ cells; C-ALL: congenital ALL; M-ALL: mixed ALL; AML: acute myeloid leukemia. Mo: mock; Pam: Pam3CSK4; Fla: flagellin; Mal: MALP2; PU: PolyU.

Interestingly, previous observations suggest that multilymphoid progenitors and more committed CD45RA^+^ lymphoid progenitor cells from normal adult bone marrow respond to TLR9 ligation by inducing IL15-R display on NK cell precursors and the quick production of functional NK cells, while their neonatal counterparts revealed no such competence (Vadillo et al., submitted). Accordingly, our results indicate that upon PolyU or CpG stimulation, the frequencies of developing CD56^+^CD11c^−^ immature NK cells significantly diminished, and a discrete CD56^+^CD11c^+^ mature NK cell population began to emerge from B-ALL or C-ALL precursors in 30-day cultures (Figure 7(a)). The cytotoxic activity of *in vitro* produced NK cells by ALL primitive progenitors was tested using nonradioactive methods, demonstrating the functional capacity of ALL-derived NK cells (Figure 7(b)). Additionally, the expression of the NKG2D molecule, an activating receptor of NK cells, was confirmed by flow cytometry (Figure 7(b)). Both cell frequencies and absolute numbers of newly formed NK cells indicate that lymphoid neoplasms have good capabilities of producing these emergent innate cells (Figures 7(c) and 7(d)). 

To understand how CpG influences the production of NK cells, we addressed the possibility of an elevated activity of Id-2, a required transcription factor in the NK differentiation pathway [38]. Substantial differences were then found when comparing Id-2 transcript levels of mock controls and CpG-stimulated cells from B-ALL (Figure 7(e)). Id-2 protein is a natural antagonist of E proteins and up-regulation of Id-2 blocks T, B, and pDC development, while NK cell production is promoted [39]. We have previously observed that normal Id-2 transcript expression is not altered upon CpG stimulation of adult BM CD34^+^ cells (Vadillo et al., submitted). In the context of leukemia, our new observations suggest that Id-2 is upregulated as a consequence of TLR9 stimulation, and consequently NK cell production is promoted in Pre-B-ALL. This was also observed in C-ALL when stimulating with TLR7/8 ligands. An inhibition of the transcription factor Pax-5 was expected since EBF-1 controls it, and Id-2 controls EBF-1. However, no such effect was achieved [40]. Additional experiments are needed to understand how transcription factors influence lineage restriction in disease and to test the functional capability of AL-derived NK cells as a result of TLR signaling. Establishing the cytotoxic or regulatory properties of NK cells that are produced when TLR signals are given in hematological neoplasms may have a crucial clinical impact.

## 4. Concluding Remarks

During hematological malignancies, including acute leukemias, Toll-like receptors provide a mechanism for producing cells of the innate immune system from early stages of hematopoietic development. To our knowledge, this is the first investigation that has been made of lymphoid precursor responses to TLR agonist ligands in the setting of acute leukemia. The findings suggest that the B lymphoid development is little influenced by infectious agents, contrasting with the promoted production of myeloid and NK cell types in response to life-threatening infections or disease-associated cell damage. Thus, TLR ligation on lymphoid progenitor cells might be exploited as a presumptive complementary strategy to improve cancer surveillance in childhood leukemia.

## Figures and Tables

**Table 1:** Patient characteristics.

Patient	Age (y)	Sex	WBC/mm^3^	Morphological diagnosis	Phenotype	BM blast infiltration (%)	Risk stratification	Risk factor
1	12	M	87,900	ALL-L2	B-ALL	88.9	HR	Leukocytosis Age
2	2	F	12300	ALL-L1	B-ALL	80	SR	—
3	7	M	13800	ALL-L1	B-ALL	96	SR	—
4	7	F	157,000	ALL-L1	B-ALL	78.1	HR	Leukocytosis
5	10	M	70,700	ALL-L1	B-ALL	80	HR	Age Leukocytosis
6	7	M	123,000	ALL-L2	B-ALL	98	HR	Leukocytosis
7	9	M	25,800	ALL-L2	B-ALL	98.5	SR	—
8	20	F	15,630	ALL-L1	B-ALL	80	HR	Age
9	13	M	211,000	ALL-L2	B-ALL	98	HR	Age Leukocytosis
10	4	M	34,220	ALL-L1	B-ALL	90	SR	—
11	4 months	F	156,800	ALL-L1	C-ALL	94	HR	Age Leukocytosis
12	7	M	17,400	ALL-L1	T-ALL	71.4	HR	T-cell phenotype
13	3	M	75,800	ALL-L1	T-ALL	89	HR	T-cell phenotype No response to initial dexamethasone treatment Leukocytosis Positive cerebrospinal fluid
14	7	M	37,300	ALL-L2	M-ALL	94.2	HR	No response to initial dexamethasone treatment
15	13	M	200,000	ALL-L2	M-ALL	68	HR	Age Leukocytosis
16	15	M	144,600	AML-M1	AML	94	HR	Leukocytosis

y: years old; WBC: white blood cell count; BM: bone marrow; M: male; F: female; B-ALL: B cell precursor acute lymphoblastic leukemia; T-ALL: T cell acute lymphoblastic leukemia; M-ALL: mixed acute leukemia; AML: Acute myeloid leukemia; C-ALL: congenital leukemia; HR: high risk; SR: standard risk.
