# Expression of Concern on “TLR Stimulation of Bone Marrow Lymphoid Precursors from Childhood Acute Leukemia Modifies Their Differentiation Potentials”

**DOI:** 10.1155/2021/3750480

**Published:** 2021-01-23

**Authors:** BioMed Research International

*BioMed Research International* would like to express concern with the article titled “TLR Stimulation of Bone Marrow Lymphoid Precursors from Childhood Acute Leukemia Modifies Their Differentiation Potentials” [1], due to concerns about potential image duplication and manipulation in Figures 1 and 7. Details of the concerns, as first raised on PubPeer [2], are as follows:

**1. Figure 1(a):**

–The eighth band in TLR-2 appears to be similar to the eighth bands in TLR-4 and TLR-6.

–The ninth and eleventh bands in TLR-2 appear to be similar.

–The fourteenth bands in TLR-2 and TLR-4 appear to be similar.

–The fourteenth bands in TLR-5, TLR-8 and TLR-9 appear to be the same.

–The ninth and eleventh bands in TLR-7 appear to be similar.

–The first band in TLR-8 appears to be similar to the first and tenth bands in TLR-9.

–The seventh band in TLR-8 appears to be similar to the seventh and the twelfth bands in TLR-10.

–There appears to be undeclared splicing in the seventh band in TLR-2, the second and eleventh bands in TLR-4, the second, seventh, tenth and twelfth bands in TLR-5, the twelfth band in TLR-6, the twelfth band in TLR-9, and the thirteenth band in TLR-10.

–In the 18S panel, bands 1-2, 3-4, and 7-8 appear to be similar.

**2. Figure 7(e):**

–In PAX-5, there appears to be undeclared splicing between the bands.

–EBP*α* and 18s are similar, when panel 4 is rotated 180 degrees.

Additionally, in Figure 5(a) for PreB-ALL, the LPS and Flag images are the same and the PolyU and CpG images are the same. This error was introduced during the redrawing of the figures in the production process.

The authors provided the underlying images and raw data for all the figures, however they did not sufficiently address the concerns raised. We have asked the institution to formally investigate.
